# Changes in the network temperature of posttraumatic stress disorder symptoms among children and adolescents following earthquakes

**DOI:** 10.1017/S0033291726105303

**Published:** 2026-07-30

**Authors:** Liying Zhang, Yi Jiang, Mingxiao Liu, Aiyi Liu, XinChun Wu, Hao Yuan, Wenchao Wang

**Affiliations:** 1 Beijing Key Laboratory of Applied Experimental Psychology, National Demonstration Center for Experimental Psychology Education (Beijing Normal University), Faculty of Psychology, Beijing Normal University, Beijing 100875, China; 2 Bay Area School of Applied Psychological Sciences, Beijing Normal University at Zhuhai, Guangdong 519087, China; 3 CAS Key Laboratory of Mental Health, Institute of Psychology, Chinese Academy of Sciences, Beijing 100101, China; 4 Pingshan Foreign Languages School, Shenzhen, Guangdong 518000, China

**Keywords:** adolescents, children, earthquake, network temperature, posttraumatic stress disorder

## Abstract

**Background:**

Posttraumatic stress disorder (PTSD) symptoms are among the most prevalent mental health sequelae in children and adolescents following earthquakes. A recently introduced metric under network theories of psychopathology, network temperature, quantifies the global stability of a symptom network.

**Methods:**

To examine the developmental trajectory of network temperature in children and adolescents after earthquakes, we established eight prospective cohorts (2–4 waves per cohort; total *N* = 5,794) after the Wenchuan, Yaan, and Jiuzhaigou earthquakes. We assessed PTSD symptoms across three postdisaster periods: early (≤2.5 years), middle (3.5–5.5 years), and late (8.5–10 years).

**Results:**

PTSD symptom network temperatures decreased across all eight cohorts. In the early post-Wenchuan period, adolescents declined more slowly than children. Across earthquakes, the early-period decline in children was faster after the Wenchuan earthquake than after the Yaan earthquake; for adolescents, the early-period decline rate followed the order Wenchuan < Jiuzhaigou < Yaan. Across post-earthquake periods, Wenchuan adolescents declined more slowly in the early period than in the middle/late period, whereas Yaan adolescents declined faster in the early period than in the middle period.

**Conclusions:**

Across all earthquake types and post-earthquake periods, the PTSD symptom network system became increasingly stable and resistant to change. These findings underscore the necessity of early intervention alongside efforts to prevent deterioration. However, the rate of this decline varied by sample (children versus adolescents), earthquake, and post-earthquake period. Clinically, this calls for intervention strategies that are adjusted according to these factors.

## Introduction

Earthquakes cause substantial economic losses, structural damage, population displacement, and fatalities (Centre for Research on the Epidemiology of Disasters, 2025), along with adverse psychological outcomes among survivors (Cénat, McIntee, & Blais-Rochette, 2020). Among these, posttraumatic stress disorder (PTSD) is a mental health condition particularly associated with earthquakes (Sirotich & Camisasca, 2024). PTSD arises following direct exposure (experiencing or witnessing a traumatic event) or indirect exposure (learning that a traumatic event occurred to a close relative, or experiencing repeated or extreme exposure to aversive details of the event) (American Psychiatric Association, 2022). The disorder is characterized by four symptom clusters: intrusive symptoms, avoidance, negative alterations in cognition and mood, and changes in arousal and reactivity (American Psychiatric Association, 2022). Children and adolescents are especially vulnerable to developing PTSD symptoms after earthquakes due to their dependence on adults (Blanc et al., 2020), and their symptoms often persist for up to a decade (Wang, Wu, & Zhou, 2018). Thus, understanding the long-term developmental pathological characteristics of PTSD symptoms in this population is critical.

In recent years, the network theory of psychopathology (Borsboom, 2017) has proposed that PTSD arises from interactions among its symptoms, a framework that disentangles the heterogeneity of symptoms and their interconnections to provide more precise insights into etiology and intervention (Birkeland, Greene, & Spiller, 2020; Borsboom, 2017). Although most studies have examined cross-sectional symptom networks (Birkeland, Greene, & Spiller, 2020), the severity of children’s and adolescents’ PTSD symptoms and their interconnections are known to evolve and fluctuate over time (Hong & Efferth, 2016; Liang, Cheng, Ruzek, & Liu, 2019; Ma et al., 2023; Wang, Wu, & Zhou, 2018). Therefore, scholars have argued that dynamic temporal networks offer a more robust reference, particularly those that account for within-person change characteristics (Borsboom, 2017; Bridges-Curry, 2025; Grimes et al., 2025).

Identifying network features that hold clinical significance through dynamic temporal networks is crucial. Previous research has emphasized the centrality of nodes (symptoms) within these networks (Birkeland, Greene, & Spiller, 2020; Bridges-Curry, 2025). However, this approach has several limitations. In cross-sectional networks, a symptom may appear to be central and thus a target for intervention. Yet, if this symptom is a consequence rather than a cause of other symptoms, targeting it may not lead to reductions in other symptoms (Grimes et al., 2025). Although longitudinal networks offer some degree of causal inference, the replicability of identified core PTSD symptoms remains poor due to variations in study intervals, types of trauma, and sample differences across studies (Birkeland, Greene, & Spiller, 2020; Bridges-Curry, 2025; Ma et al., 2023; Wu et al., 2025). Consequently, the clinical utility of emphasizing centrality within the complex system of psychological networks has been increasingly questioned by many researchers (Bringmann et al., 2019; Grimes et al., 2025).

Against this backdrop, researchers have begun to shift their focus from individual symptom centrality to global network indices within this complex system (Bringmann et al., 2019; Grimes et al., 2025; Öngür & Paulus, 2025; van Borkulo et al., 2023). Recently, network temperature has emerged as a novel metric for assessing the stability of psychological dynamic temporal networks (Borsboom et al., 2021; Grimes et al., 2025), which may contribute to understanding the fluctuations in PTSD symptoms among children and adolescents following earthquakes. Specifically, network temperature is primarily calculated based on the Ising model (Finnemann, Borsboom, Epskamp, & van der Maas, 2021; van Borkulo et al., 2014). In this framework, the Ising model is estimated using logistic regression analyses, where edge weights correspond to the regression coefficients obtained by iteratively regressing each symptom variable on all other symptoms. The intercept of the logistic regression represents the threshold parameter for each symptom, indicating its autonomous disposition for manifestation. The specific formula governing the probability of a given symptom configuration is presented in Equation (1):

(1) $$P\left(X=x\right)=\frac{e^{-\beta \left(-\sum_{i}\tau_{i}x_{i}-\sum_{i<j}\omega_{\mathrm{ij}}x_{i}x_{j}\right)}}{Z},$$

where $P\left(X=x\right)$ denotes the probability of the network being in a specific configuration x . The configuration X represents the particular states of all nodes in the network, with each node $x_{i}$ capable of being in one of two states (e.g., −1 or 1). The expression in the exponent, $\left(-\sum_{i}\tau_{i}x_{i}-\sum_{i<j}\omega_{\mathrm{ij}}x_{i}x_{j}\right)$ , is the Hamiltonian function, which represents the energy of a given symptom configuration. The Hamiltonian integrates the two structural components of the network: $\tau_{i}$ represents the threshold parameter for symptom i (capturing its autonomous tendency to activate), and $\omega_{\mathrm{ij}}$ represents the edge weight between symptoms i and j (quantifying their pairwise interaction). The parameter β represents the inverse temperature ( β = 1/network temperature), scaling the influence of the Hamiltonian on the probability. A high β (low temperature) indicates that the system strongly favors low-energy states with reduced randomness. Z is the partition function, which sums the numerator over all possible network configurations to ensure that the probabilities sum to 1, serving as a normalization constant.

Crucially, network temperature functions as a global scaling factor applied uniformly to the entire ‘network skeleton’—that is, to both the edge weights ( $\omega_{\mathrm{ij}}$ ) and the thresholds ( $\tau_{i}$ ). At low network temperatures (high β ), the system exhibits relatively little randomness; the influence of the skeleton is amplified, thereby constraining node states to converge toward a limited set of low-energy configurations. Taking PTSD symptoms as an example, under such low-temperature conditions, symptom expression tends to follow the network skeleton closely, and the reduced randomness implies a more stable system that is less susceptible to change. Conversely, high network temperatures (low β ) effectively scale down the influence of both the interactions and thresholds, such that random thermal fluctuations override the structural constraints. In this case, symptoms may be unstable, varying unpredictably over time, and reflecting a system that is highly susceptible to change. This high-temperature state may thus represent a window of opportunity for modifying the PTSD symptom system: well-timed positive interventions are more likely to take effect, while adverse influences may also exact a greater toll (Grimes et al., 2025).

This study aimed to investigate changes in PTSD symptom network temperature among children and adolescents across distinct post-earthquake periods, to elucidate how the stability of their PTSD symptom network system unfolds over time. Leveraging eight prospective cohorts (*N* = 5,794; 2–4 assessment waves) established after the Wenchuan, Yaan, and Jiuzhaigou earthquakes, we examined PTSD network temperature trajectories stratified by three post-disaster periods: early (≤2.5 years), middle (3.5–5.5 years), and late (8.5–10 years). Based on our findings, we aimed to advance the understanding of the longitudinal psychopathology of PTSD in children and adolescents after earthquakes and carry implications for clinical practice.

## Methods

### Participants and procedure

Our study was conducted following three major earthquakes in Wenchuan, Yaan, and Jiuzhaigou (see Supplementary Table S1 for detailed information about the three earthquakes). To understand the mental health status of children (primary school students) and adolescents (middle school students) at various stages following earthquakes and to provide psychological assistance accordingly, we conducted eight cohort surveys following three earthquakes. Each cohort comprised several cross-sectional studies (2–4 waves) conducted in fixed schools in severely affected areas. It should be noted that, to gain a comprehensive understanding of the psychological status of each child and adolescent in the disaster areas, the subsequent waves of surveys did not strictly adhere to a longitudinal tracking design. Specifically, each wave of the cohort recruited participants from the disaster areas, irrespective of their involvement in previous surveys.

Initially, we established contact with local education authorities to inform them of the research objectives and methodologies. We also assured them that additional psychological support services would be available if needed. Subsequently, we identified several primary and middle schools in the areas most affected by the earthquake and established a collaboration with them. The study received ethical approval from the Human Research Ethics Committee of the first author’s institution. Moreover, written informed consent was obtained from all participants and their parents.

Our analysis included a total of eight cohorts. The assessment time points for each wave of every cohort were detailed in Table 1. Aligning with the previous study (Wang, Li, Yuan, & Wu, 2024), we stratified the cohorts into three post-earthquake periods: early (≤2.5 years; Cohorts 1, 4, 5, 7), middle (3.5–5.5 years; Cohorts 2, 6), and late (8.5–10 years; Cohort 3).

**Table 1.** Sample sizes per cohort Table 1. long description.

Middle school students after the Wenchuan earthquake (Cohort 1)	Time points after the earthquake	1 year later	1.5 years later	2 years later	2.5 years later
	Sample size of cross-sectional studies	1253	1224	900	487
	The sample size of participants who finished at least three waves	602			
	The sample size of participants who finished all waves	145			
Middle school students after the Wenchuan earthquake (Cohort 2)	Time points after the earthquake	3.5 years later	4.5 years later	5.5 years later	
	Sample size of cross-sectional studies	364	331	161	
	The sample size of participants who finished at least two waves	332			
	The sample size of participants who finished all waves	137			
Middle school students after the Wenchuan earthquake (Cohort 3)	Time points after the earthquake	8.5 years later	9.5 years later	10 years later	10.5 years later
	Sample size of cross-sectional studies	2341	1648	1565	1031
	The sample size of participants who finished at least three waves	1227			
	The sample size of participants who finished all waves	473			
Primary school students after the Wenchuan earthquake (Cohort 4)	Time points after the earthquake	1 year later	1.5 years later		
	Sample size of cross-sectional studies	1511	984		
	The sample size of participants who finished at least two waves	753			
	The sample size of participants who finished all waves	753			
Middle school students after the Yaan earthquake (Cohort 5)	Time points after the earthquake	0.5 year later	1 year later	1.5 years later	2 years later
	Sample size of cross-sectional studies	625	594	535	813
	The sample size of participants who finished at least three waves	503			
	The sample size of participants who finished all waves	168			
Middle school students after the Yaan earthquake (Cohort 6)	Time points after the earthquake	3.5 years later	4.5 years later	5.5 years later	
	Sample size of cross-sectional studies	1420	1104	646	
	The sample size of participants who finished at least two waves	1019			
	The sample size of participants who finished all waves	300			
Primary school students after the Yaan earthquake (Cohort 7)	Time points after the earthquake	0.5 year later	1 year later	1.5 years later	2 years later
	Sample size of cross-sectional studies	303	296	308	304
	The sample size of participants who finished at least three waves	303			
	The sample size of participants who finished all waves	132			
Middle school students after the Jiuzhaigou earthquake (Cohort 8)	Time points after the earthquake	10 months later	22 months later	27 months later	
	Sample size of cross-sectional studies	1241	1319	624	
	The sample size of participants who finished at least two waves	1055			
	The sample size of participants who finished all waves	190			

The sample sizes for each cohort are shown in Table 1, and the number of participants who completed all waves in each cohort is also presented. It should be noted that some cohorts comprised relatively small samples of participants who completed all assessment waves, which may lead to unstable results in network analysis (Isvoranu & Epskamp, 2023). Therefore, for cohorts with three or more waves, we included cases that participated in at least the total wave counts minus 1 and imputed the missing data (see the Methods section for details) to form our primary analytic sample (sample size was shown in Table 1). However, this approach to sample selection and imputation might introduce some degree of bias. Thus, following the recommendations of prior research (Grimes et al., 2025), we also conducted analyses on the subsample of participants who completed all waves across all cohorts (sample size see Table 1) to ensure the robustness of our findings.

### Measures

The Child PTSD Symptoms Scale for DSM-IV (CPSS; Foa, Johnson, Feeny, & Treadwell, 2001) was employed to assess PTSD symptoms in Cohorts 1, 2, 4, 5, and 7. This scale comprises 17 items, rated on a 4-point scale ranging from 0 (*never*) to 3 (*always*), and includes three subscales: intrusions, avoidance, and hyperarousal. The scale has demonstrated good reliability and validity among Chinese children and adolescents who experienced an earthquake (Zhen, Zhou, & Wu, 2019). In the present study, the Cronbach’s α coefficients for the CPSS ranged from 0.81 to 0.93 across all waves.

For Cohorts 3, 6, and 8, PTSD symptoms were measured using the PTSD Checklist for the DSM-5 (PCL-5), developed by Weathers et al. (2013). This checklist consists of 20 items and four subscales: intrusions, negative cognition and emotion alteration, avoidance, and hyperarousal. Each item is rated on a 5-point Likert scale, ranging from 0 *(completely disagree)* to 4 *(completely agree).* However, to maintain consistency with the 0–3 scoring range historically used in Wenchuan and Yaan earthquake datasets, Cohort 3 and Cohort 6 used a modified 0–3 scoring (rather than the standard 0–4), whereas Cohort 8 retained the original 0–4 scoring of the PCL-5. The reliability and validity of this scale have been previously established in studies involving post-earthquake Chinese children and adolescents (Zhou, Wu, & Zhen, 2017). In the current study, the Cronbach’s α internal consistency coefficients for this scale ranged from 0.84 to 0.92 across all waves.

### Data analysis

All data analyses in this study were conducted using R version 4.3.1. The primary analysis code is available at https://osf.io/4jrst (Zhang, 2026).

#### Symptom cleaning and multiple imputation

The symptom data for all eight cohorts were scored on a 4- or 5-point Likert scale (where the lowest value represents the absence of symptoms, and the other values indicate varying degrees of symptom severity). In line with the Ising model’s conceptualization of symptom ‘on’/‘off’ states, we recoded the lowest value (0, indicating no symptoms) as −1, and all other values as 1 (Grimes et al., 2025). Subsequently, following the approach used in prior research (Grimes et al., 2025), we applied multiple imputation by chained equations with predictive mean matching using the *mice* package in R. The number of imputed datasets was set to the percentage of missing data, with 20 iterations for each imputation. The final analytic dataset was obtained by aggregating the imputed datasets and selecting the modal symptom combination as the pooled point estimate. As mentioned earlier, to assess the impact of both attrition and imputation, we estimated the networks and extracted temperature changes for all cohorts using the maximum-likelihood estimator, based on the nonimputed case dataset.

#### Network estimation and network temperature

To estimate PTSD symptom networks, we employed the multigroup Ising model with binary variables, as described by Finnemann et al. (2021) and Grimes et al. (2025). Following the guidelines of Grimes et al. (2025), we utilized the Ising model function of the *psychonetrics* package in R to independently estimate network models at different time points (waves). In this framework, edge weights are denoted by the parameter omega, thresholds by tau, and inverse temperature by beta. For each cohort, we estimated four network models using an iterative approach with increasing constraints: (1) fully saturated with all parameters free; (2) set omega equal between groups for equal edges (structure) over time; (3) set omega and tau equal between groups for equal edges and external information (thresholds) over time, respectively; and (4) set omega, tau and beta equal between groups for equal edges, thresholds and temperature over time, respectively. For each of these four models, we also tested dense network (fully connected) and sparse network (pruned edges) structures, resulting in eight models estimated for each cohort. This approach has been extensively detailed in previous Ising model methodologies (Borsboom et al., 2021).

According to the guidelines of Grimes et al. (2025), the optimal model was selected based on model fit criteria, including low Akaike Information Criterion, low Bayesian Information Criterion, root mean square error of approximation (RMSEA) < 0.05, and chi-square test *p* values (*α* = 0.05). The RMSEA was calculated by comparing the subsequent models with increasing constraints to the fully connected (saturated) Ising model, which has zero degrees of freedom for pairwise interactions. This method is analogous to structural equation modelling. We conducted pairwise nested comparisons between dense and sparse networks, with Model 1 serving as the reference model.

Once the ideal Ising model was identified, network temperature was calculated as the inverse of the beta parameter (inverse temperature) in the network formula (for detailed formulas and derivations, see Grimes et al., 2025). At wave 1 of each cohort, beta (inverse temperature) was not identifiable and was thus fixed at 1 (resulting in a network temperature of 1). Adding equality constraints for subsequent time points allowed for the identification of network temperature from wave 2 onward. Consequently, network temperature could increase or decrease relative to this initial value. Finally, following the approach of Grimes et al. (2025), we computed the entropy S for each network using the formula

(2) $$S=-\sum_{x}Ρ\left(x\right)\log P\left(x\right),$$

where $P\left(x\right)$ has the same meaning as in Equation (1). In this calculation, entropy incorporates both the network temperature and the network skeleton (i.e., edge weights and thresholds). Unlike network temperature, which reflects the degree of randomness (or instability) of the system and determines the extent to which network states conform to the skeleton, entropy places greater emphasis on the behavior of symptom nodes and quantifies the stability of the system’s states. Higher entropy indicates lower stability, meaning that symptom node states tend toward disorganization rather than consistency.

## Results

The demographic information of the participants and the descriptive statistics of PTSD symptoms were presented in Table 2. In detail, Supplementary Figure S1 depicts the overall level of PTSD symptoms, and Supplementary Figure S2 illustrates their incidence rate (coded binarily within the Ising model framework). Overall, distinct temporal trends in PTSD symptom severity were observed across cohorts, including overall decreases, increases, initial decreases followed by increases, and initial increases followed by decreases. For all eight cohorts, the optimal model fit was achieved using sparse network structures with fixed edge weights (or both fixed edge weights and thresholds) while permitting external fields and temperature to vary over time. The detailed model fit results, corresponding to models with increasing levels of constraint, were reported in Supplementary Tables S2 and S3.

**Table 2.** Participant demographics and PTSD symptom statistics Table 2. long description.

Middle school students after the Wenchuan earthquake (Cohort 1)	Time points after the earthquake	1 year later	1.5 years later	2 years later	2.5 years later
	Descriptive statistics of PTSS (*M* ± *SD*)	13.98 ± 9.42	13.73 ± 8.99	14.65 ± 8.74	14.07 ± 8.46
	Descriptive statistics of age at wave 1 (*M* ± *SD*)	14.70 ± 1.72			
	Descriptive statistics of sex at wave 1 (percentage of females)	61.0%			
Middle school students after the Wenchuan earthquake (Cohort 2)	Time points after the earthquake	3.5 years later	4.5 years later	5.5 years later	
	Descriptive statistics of PTSS (*M* ± *SD*)	12.95 ± 7.01	13.46 ± 7.63	13.16 ± 7.49	
	Descriptive statistics of age at wave 1 (*M* ± *SD*)	15.07 ± 1.65			
	Descriptive statistics of sex at wave 1 (percentage of females)	53.5%			
Middle school students after the Wenchuan earthquake (Cohort 3)	Time points after the earthquake	8.5 years later	9.5 years later	10 years later	10.5 years later
	Descriptive statistics of PTSS (*M* ± *SD*)	13.03 ± 8.32	12.96 ± 8.76	12.95 ± 10.05	12.09 ± 9.09
	Descriptive statistics of age at wave 1 (*M* ± *SD*)	14.12 ± 2.28			
	Descriptive statistics of sex at wave 1 (percentage of females)	60.0%			
Primary school students after the Wenchuan earthquake (Cohort 4)	Time points after the earthquake	1 year later	1.5 years later		
	Descriptive statistics of PTSS (*M* ± *SD*)	13.22 ± 7.07	10.60 ± 6.93		
	Descriptive statistics of age at wave 1 (*M* ± *SD*)	10.97 ± 0.98			
	Descriptive statistics of sex at wave 1 (percentage of females)	48.9%			
Middle school students after the Yaan earthquake (Cohort 5)	Time points after the earthquake	0.5 year later	1 year later	1.5 years later	2 years later
	Descriptive statistics of PTSS (*M* ± *SD*)	14.21 ± 8.34	14.43 ± 8.05	13.68 ± 8.93	12.81 ± 7.50
	Descriptive statistics of age at wave 1 (*M* ± *SD*)	14.51 ± 1.93			
	Descriptive statistics of sex at wave 1 (percentage of females)	56.1%			
Middle school students after the Yaan earthquake (Cohort 6)	Time points after the earthquake	3.5 years later	4.5 years later	5.5 years later	
	Descriptive statistics of PTSS (*M* ± *SD*)	12.87 ± 9.62	12.73 ± 9.41	14.00 ± 10.87	
	Descriptive statistics of age at wave 1 (*M* ± *SD*)	13.93 ± 1.65			
	Descriptive statistics of sex at wave 1 (percentage of females)	56.1%			
Primary school students after the Yaan earthquake (Cohort 7)	Time points after the earthquake	0.5 year later	1 year later	1.5 years later	2 years later
	Descriptive statistics of PTSS (*M* ± *SD*)	11.75 ± 7.15	12.48 ± 8.20	11.39 ± 8.06	8.63 ± 7.53
	Descriptive statistics of age at wave 1 (*M* ± *SD*)	9.73 ± 0.91			
	Descriptive statistics of sex at wave 1 (percentage of females)	49.8%			
Middle school students after the Jiuzhaigou earthquake (Cohort 8)	Time points after the earthquake	10 months later	22 months later	27 months later	
	Descriptive statistics of PTSS (*M* ± *SD*)	28.91 ± 13.79	25.36 ± 14.28	26.08 ± 14.05	
	Descriptive statistics of age at wave 1 (*M* ± *SD*)	14.60 ± 1.54			
	Descriptive statistics of sex at wave 1 (percentage of females)	59.0%			

**Figure 1.**
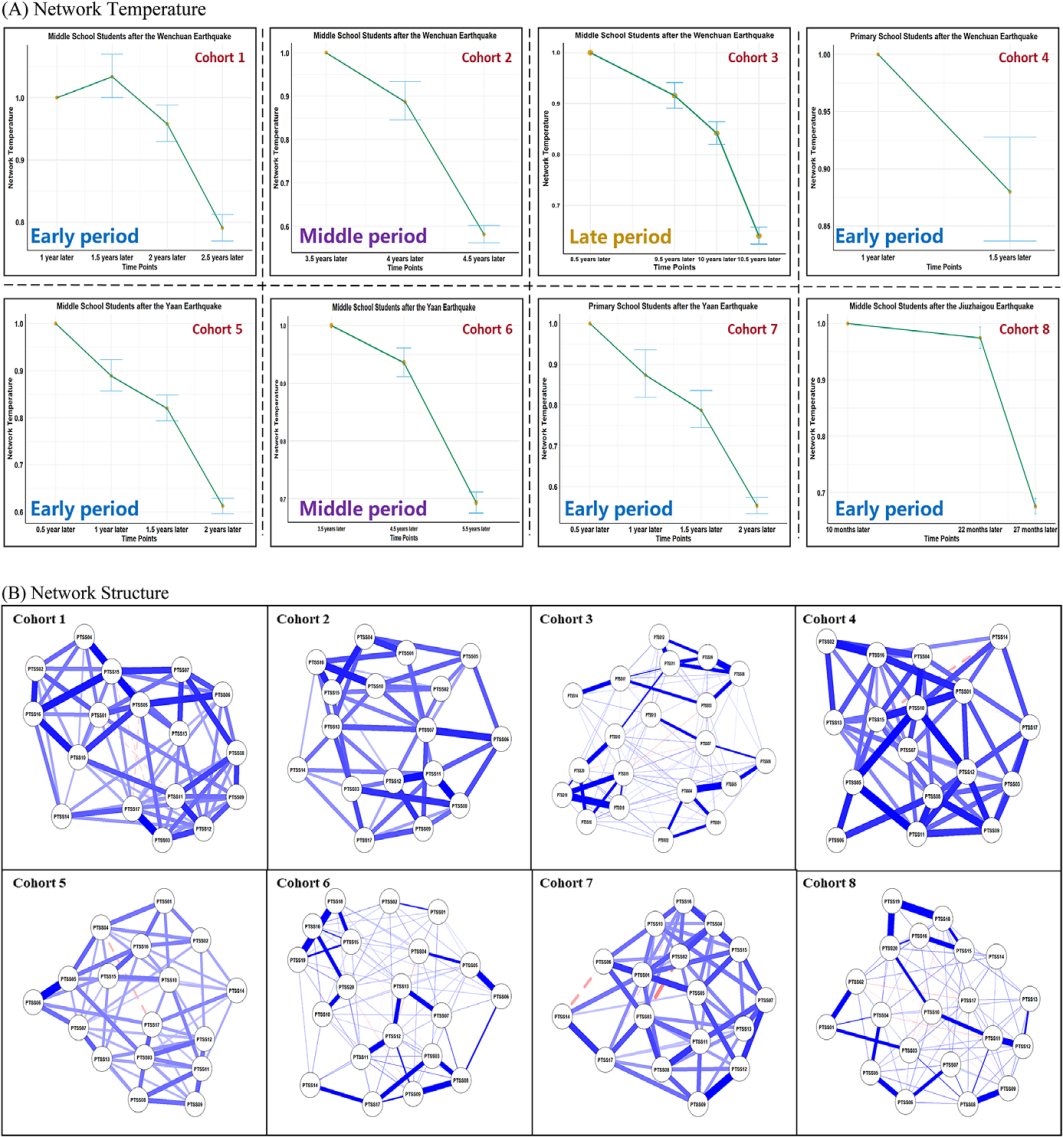
PTSD network temperature after earthquakes*. Note:* The error bars indicate 95% confidence intervals. See Supplementary Table S6 for node names. Solid lines denote positive edges; dashed lines denote negative edges. When estimating network temperature changes across all cohorts, the Ising model with edge weights constrained to be equal across waves within each cohort provided the best fit to the data (see Supplementary Table S2). Consequently, the displayed network structure is representative of all waves, as the edges are identical across time points. Figure 1. long description.

Across all eight cohorts, network temperature exhibited a declining trend, except for a slight increase in Cohort 1 from Waves 1 to 2 (see Table 3 and Figure 1). A similar pattern of network temperature reduction was observed in the non-imputed case dataset (see Supplementary Table S4 and Figure S3), indicating robust findings. In both datasets, the entropy values for each network are presented in Supplementary Table S5. Mirroring the temperature trajectories, entropy also showed a broadly decreasing trend within each cohort.

**Table 3.** PTSD network temperature after earthquakes Table 3. long description.

Middle school students after the Wenchuan earthquake (Cohort 1)	Time points after the earthquake	1 year later	1.5 years later	2 years later	2.5 years later
	Network temperature [95% CI]	1	1.03 [1.00, 1.07]	0.96 [0.93, 0.99]	0.79 [0.77, 0.81]
	Average annual decline in network temperature (V_T_)	0.14			
Middle school students after the Wenchuan earthquake (Cohort 2)	Time points after the earthquake	3.5 years later	4.5 years later	5.5 years later	
	Network temperature [95% CI]	1	0.89 [0.84, 0.93]	0.58 [0.56, 0.60]	
	Average annual decline in network temperature (V_T_)	0.21			
Middle school students after the Wenchuan earthquake (Cohort 3)	Time points after the earthquake	8.5 years later	9.5 years later	10 years later	10.5 years later
	Network temperature [95% CI]	1	0.92 [0.89, 0.94]	0.84 [0.82, 0.86]	0.64 [0.62, 0.66]
	Average annual decline in network temperature (V_T_)	0.18			
Primary school students after the Wenchuan earthquake (Cohort 4)	Time points after the earthquake	1 year later	1.5 years later		
	Network temperature [95% CI]	1	0.88 [0.84, 0.93]		
	Average annual decline in network temperature (V_T_)	0.24			
Middle school students after the Yaan earthquake (Cohort 5)	Time points after the earthquake	0.5 year later	1 year later	1.5 years later	2 years later
	Network temperature [95% CI]	1	0.89 [0.86, 0.92]	0.82 [0.79, 0.85]	0.61 [0.60, 0.63]
	Average annual decline in network temperature (V_T_)	0.26			
Middle school students after the Yaan earthquake (Cohort 6)	Time points after the earthquake	3.5 years later	4.5 years later	5.5 years later	
	Network temperature [95% CI]	1	0.94 [0.91, 0.96]	0.69 [0.68, 0.71]	
	Average annual decline in network temperature (V_T_)	0.15			
Primary school students after the Yaan earthquake (Cohort 7)	Time points after the earthquake	0.5 year later	1 year later	1.5 years later	2 years later
	Network temperature [95% CI]	1	0.87 [0.82, 0.94]	0.79 [0.74, 0.84]	0.55 [0.53, 0.57]
	Average annual decline in network temperature (V_T_)	0.30			
Middle school students after the Jiuzhaigou earthquake (Cohort 8)	Time points after the earthquake	10 months later	22 months later	27 months later	
	Network temperature [95% CI]	1	0.97 [0.96, 0.99]	0.68 [0.66, 0.69]	
	Average annual decline in network temperature (V_T_)	0.23

Further, we conducted a detailed comparison of the differences in these declining trends. First, the specific pattern of decline within each cohort (e.g., rapid initial decline followed by slower decline, or vice versa) varied between the imputed and non-imputed case datasets. Therefore, we refrained from interpreting these specific patterns. In addition to the data presented in Tables 3 and Supplementary Table S4, we calculated the average annual decline in network temperature for each cohort (denoted as V_T_) to support the following descriptions of our results. We focused primarily on findings in which V_T_ trends were consistent between the imputed and nonimputed case datasets (e.g., in both datasets, the V_T_ for children in the early period after the Wenchuan earthquake was greater than that for adolescents in the same period) to ensure the robustness of these results.

When examining differences between children and adolescents, we compared the corresponding cohorts from Wenchuan and Yaan earthquakes: Cohort 4 versus the first two waves of Cohort 1 (network temperature increased for the imputed case dataset), and Cohort 7 versus Cohort 5. The results revealed consistent findings between imputed and nonimputed case datasets for the Wenchuan earthquake in the early period: children exhibited a faster rate of decline than adolescents. However, this consistency between imputed and non-imputed case datasets was not observed for the Yaan earthquake.

Next, we compared differences between earthquakes. For the child samples, comparisons were only possible in the early period following the Wenchuan and Yaan earthquakes (Cohort 4 versus Cohort 7), as the other cohorts did not include child samples. The results indicated that children exposed to the Wenchuan earthquake exhibited a faster decline rate than those exposed to the Yaan earthquake. For the adolescent samples, in the early period, adolescents exposed to the Wenchuan earthquake (Cohort 1) exhibited a slower decline rate than those exposed to the Jiuzhaigou earthquake (Cohort 8), and even slower than those exposed to the Yaan earthquake (Cohort 5), and this pattern was consistent across both imputed and nonimputed case datasets. In the middle period, the relative magnitude of the decline rate for adolescents exposed to the Wenchuan earthquake (Cohort 2) compared to those exposed to the Yaan earthquake (Cohort 6) was inconsistent between the imputed and non-imputed case datasets. In the late period, only a Wenchuan cohort was available, precluding comparisons between earthquakes.

Regarding comparisons across periods, cohorts in the middle and late periods, as well as the Jiuzhaigou cohort, consisted solely of adolescent samples; therefore, our comparisons of differences across periods focused primarily on adolescents to eliminate confounding by sample differences. We focused on adolescents exposed to the Wenchuan and Yaan earthquakes because Jiuzhaigou only had data for the early period. For adolescents exposed to the Wenchuan earthquake, the decline rate during the early period (Cohort 1) was slower than during both the middle period (Cohort 2) and the late period (Cohort 3), and this pattern was consistent across imputed and non-imputed case datasets. However, the relationship between the middle and late periods was inconsistent between the imputed and nonimputed case datasets. For adolescents exposed to the Yaan earthquake, the decline rate during the early period (Cohort 5) was faster than during the middle period (Cohort 6), and this pattern was consistent across both imputed and nonimputed case datasets.

## Discussion

Based on recent developments in network theory emphasizing global network characteristics and the novel metric of network temperature (Bringmann et al., 2019; Grimes et al., 2025; Öngür & Paulus, 2025; van Borkulo et al., 2023), we examined changes in PTSD symptom network temperature across eight child–adolescent cohorts assessed at different post-earthquake periods to elucidate shifts in the stability of the PTSD symptom system. Our findings advance our understanding of the longitudinal psychopathology of PTSD in children and adolescents after earthquakes and carry implications for clinical practice.

Our results revealed that although the trends in mean PTSD symptom levels and their variance across the eight cohorts varied inconsistently over time and between earthquakes, a key consistent pattern emerged: network temperature decreased universally in all cohorts. This decline reflects reduced randomness in the PTSD symptom network system; that is, node states become less erratic and more strongly governed by the network skeleton (i.e., the edge weights and thresholds), leading to more entrenched, stable symptom presentations (Grimes et al., 2025). In other words, the PTSD symptom system becomes increasingly rigid and resistant to change. We speculate that this pattern might be attributable to the dynamics of trauma-related memory strength. Over time, trauma memories of the earthquake might gradually fade, yet early earthquake-related cues remain especially salient in the brain, sustaining a PTSD symptom system that is initially highly active and changeable (Ehlers & Clark, 2000; Wu, Zhou, Wang, & Tian, 2018). Such a change can unfold in two directions. On the one hand, individuals might adopt a range of positive coping strategies, which, together with timely interventions (e.g., provision of survival resources and basic psychological support), might facilitate the adaptive integration of trauma memories and thereby alleviate PTSD symptoms (Ehlers & Clark, 2000; Tedeschi & Calhoun, 2004; Wu et al., 2018). On the other hand, maladaptive coping strategies and external risk factors (such as secondary trauma exposure) might promote negative integration, including negative appraisals of the trauma or its aftermath, which in turn exacerbates PTSD symptoms (Ehlers & Clark, 2000; Elwood, Mott, Lohr, & Galovski, 2011; Wu et al., 2018). Hence, when network temperature is high, that is, immediately after an earthquake and before the system has stabilized, there is both an urgent need to implement positive interventions and a concurrent need to prevent further deterioration.

These findings regarding changes in PTSD symptom network properties can be mapped onto the established trajectories of PTSD symptom levels following earthquakes. Previous studies have identified four distinct symptom trajectories: chronic dysfunction (persistently high symptoms), delayed dysfunction (initially low symptoms followed by a subsequent increase), resilience (consistently low symptoms), and recovery (initially high symptoms followed by a decline) (Cheng et al., 2019; Ma et al., 2023). The chronic dysfunction and resilience groups, which are characterized by greater symptom stability, are typically associated with lower network temperature; conversely, the delayed dysfunction and recovery groups, which exhibit more dynamic symptom changes, tend to be associated with higher network temperature (Grimes et al., 2025). The observed decline in network temperature among children and adolescents after earthquakes was associated with an increased prevalence of the chronic dysfunction and resilience groups, alongside a decrease in the delayed dysfunction and recovery groups (Grimes et al., 2025). The extent of proportional shifts and changes in these groups was likely associated with the specific trajectories of PTSD symptom levels in each cohort (see Supplementary Figures S1 and S2).

Beyond their connection to PTSD symptom levels, changes in network temperature are also linked to PTSD symptom heterogeneity, a central focus of network theory (Borsboom, 2017), and thus carry additional clinical implications. Even when total symptom levels remain constant, symptom subgroups or profiles might diverge—for instance, through an increase in intrusive symptoms alongside a decrease in hyperarousal symptoms—reflecting growing heterogeneity among symptom profiles and dynamic interchange between them over time (Boasso, Steenkamp, Larson, & Litz, 2016; Goodman-Williams, Clark, Campbell, & Ullman, 2024). The recognition of PTSD subtypes, such as the dissociative subtype, and the distinction of complex PTSD further underscore the clinical relevance of symptom heterogeneity (Ben‐Ezra et al., 2018; Friedman, 2013).

Therefore, a decline in network temperature, which signifies reduced randomness of the symptom system, might be associated with reduced diversity in PTSD symptom profiles across individuals. This is further corroborated by the concurrent decline in network entropy across all cohorts (where network entropy measures state instability, with higher entropy signifying greater diversity in symptom expression). Initially, children and adolescents within each cohort likely exhibited the most diverse symptom profiles—for example, some predominantly displayed intrusion symptoms, others primarily avoidance symptoms (Boasso et al., 2016; Goodman-Williams et al., 2024). Over time, an increasing number of individuals tended to converge on more homogeneous symptom profiles, coalescing into one or a few groups that shared similar symptom features. Although early intervention remains crucial (Grimes et al., 2025; McElroy, Napoleone, Wolpert, & Patalay, 2019), the high symptom heterogeneity present when network temperature is elevated was associated with increased cost and complexity of delivering personalized, person‑centered interventions. Therefore, further research is needed to examine how elevated network temperature relates to person‑centered treatment planning.

We further examined differences in the rate of network temperature reduction across samples (children versus adolescents), post-earthquake periods, and different earthquakes. These results might not be entirely robust; therefore, the following interpretations should be viewed with caution. The rate of decrease was faster among children in the early post-Wenchuan period than among adolescents and faster than that of children in the early post-Yaan period. This suggests that children’s PTSD symptom systems are more susceptible to change and stabilize more rapidly, especially after a more severe earthquake. One possible explanation is that children receive more prioritized protection after a major earthquake (Bhadra, 2022), and their relatively lower direct trauma exposure compared with adolescents might result in fewer and less consolidated trauma memories (Ehlers & Clark, 2000). Consequently, the randomness of the symptom system diminishes more quickly. A complementary explanation is that children’s trauma memories, particularly after a severe earthquake, are more likely to be latent and implicit in nature (e.g., encoded as bodily cues) rather than integrated into verbally accessible autobiographical memories (McCrory & Viding, 2015). While these implicit memories might not directly perturb self-reported PTSD symptoms in the early period, their influence might only become manifest in the middle or late period—a period not covered by our current data.

Conversely, the rate of network temperature decline among adolescents in the early post-Wenchuan period was slower than the early post-earthquake rates among adolescents after the Jiuzhaigou and Yaan earthquakes and slower than the rate observed during the middle/late post-Wenchuan period. Notably, the early post-Yaan adolescent decline rate was faster than that during the middle period. This pattern implies that, although the overall trend is one of decreasing network temperature, adolescents who experienced the more severe Wenchuan earthquake maintain a PTSD system that remains relatively random and variable for a longer time, with a more accelerated drop occurring only in the middle/late period. This prolongation might be due to more profoundly encoded trauma memories (Ehlers & Clark, 2000), which keep the associated PTSD symptom system in a persistently heightened state of activation, compounded by the fact that adolescents are navigating a critical developmental stage already characterized by heightened uncertainty (such as rapid physical, cognitive, and emotional changes; Cisler & Herringa, 2021).

Importantly, these findings call for a dialectical interpretation. The rapid early decline in network temperature among children after a severe earthquake might imply a potentially narrow intervention window. Yet, during this very period, PTSD symptom profiles tend to be highly heterogeneous, which might require interventions tailored to symptom subtypes (Boasso et al., 2016; Goodman-Williams et al., 2024). Moreover, it is essential to remain vigilant against the delayed impact of latent, implicit trauma representations, which might only surface at later developmental stages (McCrory & Viding, 2015). Accordingly, even children whose early symptoms subside quickly after a severe earthquake should receive longitudinal monitoring, and caregivers should be equipped to recognize and respond to delayed behavioral or somatic expressions of trauma. Conversely, the slower early decline in network temperature among adolescents after a severe earthquake offers a more extended intervention window. However, the prolonged period of a random and highly susceptible symptom network also carries a substantial risk of deterioration. Extra caution must be directed toward secondary trauma exposure in the early post-earthquake period (Elwood et al., 2011), alongside attention to the normative developmental crises of adolescence (such as emotion dysregulation, academic pressure, and interpersonal difficulties) that render this population particularly vulnerable (Cisler & Herringa, 2021). Nonetheless, while the above discussion highlights the urgency of intervention in the early post-earthquake period, especially after severe earthquakes (e.g., Wenchuan), this does not imply that intervention and deterioration prevention become less important following milder earthquakes (e.g., Yaan, Jiuzhaigou) or during the middle and late post-earthquake periods. Sustained, school–family–community collaborative psychological screening and support remain essential for all children and adolescents who have experienced earthquakes.

However, this study has several limitations. First, because our study involved multiple cohorts from different earthquakes, the assessment instruments used were not uniform. Across the cohorts, the number of PTSD symptoms measured varied (17 or 20), and the rating scales differed (0–4 or 0–3). Nevertheless, consistent findings were obtained from sensitivity analyses. Second, the cohorts did not cover all possible time points following each earthquake, preventing the formation of continuous long-term cohorts. Consequently, although network temperature decreased across all observed cohorts, we cannot definitively infer a universal decline in PTSD symptom network temperature for children and adolescents following earthquakes. Third, while prior research posits that periods of high network temperature represent optimal intervention windows (Grimes et al., 2025), this instability may theoretically also indicate heightened vulnerability to risk factors. Moreover, the concomitant high individual heterogeneity in symptoms during such periods poses significant challenges for personalized intervention. Future intervention studies are needed to clarify the clinical utility of network temperature for guiding interventions. Furthermore, this study encountered substantial participant attrition during follow-up. While we performed sensitivity analyses comparing complete cases and imputed datasets following established procedures (Grimes et al., 2025), the development of more sophisticated missing data methods remains essential for longitudinal network analysis.

## Supporting information

10.1017/S0033291726105303.sm001 Zhang et al. supplementary material Zhang et al. supplementary material

## Data Availability

The data supporting the findings of this study are available from the corresponding author upon reasonable request. The primary analysis code is available at https://osf.io/4jrst.

## Supplementary material

The supplementary material for this article can be found at http://doi.org/10.1017/S0033291726105303.

## Long descriptions

### Table 1. Long description

The table is organized into eight cohorts based on student type and earthquake event.

* Cohort 1: Middle school students after Wenchuan earthquake. Cross-sectional samples at 1, 1.5, 2, and 2.5 years were 1253, 1224, 900, and 487 respectively. 602 finished at least three waves; 145 finished all waves.

* Cohort 2: Middle school students after Wenchuan earthquake. Cross-sectional samples at 3.5, 4.5, and 5.5 years were 364, 331, and 161. 332 finished at least two waves; 137 finished all waves.

* Cohort 3: Middle school students after Wenchuan earthquake. Cross-sectional samples at 8.5, 9.5, 10, and 10.5 years were 2341, 1648, 1565, and 1031. 1227 finished at least three waves; 473 finished all waves.

* Cohort 4: Primary school students after Wenchuan earthquake. Cross-sectional samples at 1 and 1.5 years were 1511 and 984. 753 finished at least two waves and all waves.

* Cohort 5: Middle school students after Yaan earthquake. Cross-sectional samples at 0.5, 1, 1.5, and 2 years were 625, 594, 535, and 813. 503 finished at least three waves; 168 finished all waves.

* Cohort 6: Middle school students after Yaan earthquake. Cross-sectional samples at 3.5, 4.5, and 5.5 years were 1420, 1104, and 646. 1019 finished at least two waves; 300 finished all waves.

* Cohort 7: Primary school students after Yaan earthquake. Cross-sectional samples at 0.5, 1, 1.5, and 2 years were 303, 296, 308, and 304. 303 finished at least three waves; 132 finished all waves.

* Cohort 8: Middle school students after Jiuzhaigou earthquake. Cross-sectional samples at 10, 22, and 27 months were 1241, 1319, and 624. 1055 finished at least two waves; 190 finished all waves.

Navigate back to Table 1..

### Table 2. Long description

The table is divided into eight student cohorts and presents the means and standard deviations of PTSS scores and age, along with the sex ratio.

Cohort 1: Middle school students after the Wenchuan earthquake. Wave 1 age 14.70 plus or minus 1.72, 61.0 percent female. P T S S scores at 1, 1.5, 2, and 2.5 years later are 13.98 plus or minus 9.42, 13.73 plus or minus 8.99, 14.65 plus or minus 8.74, and 14.07 plus or minus 8.46 respectively.

Cohort 2: Middle school students after the Wenchuan earthquake. Wave 1 age 15.07 plus or minus 1.65, 53.5 percent female. P T S S scores at 3.5, 4.5, and 5.5 years later are 12.95 plus or minus 7.01, 13.46 plus or minus 7.63, and 13.16 plus or minus 7.49.

Cohort 3: Middle school students after the Wenchuan earthquake. Wave 1 age 14.12 plus or minus 2.28, 60.0 percent female. P T S S scores at 8.5, 9.5, 10, and 10.5 years later are 13.03 plus or minus 8.32, 12.96 plus or minus 8.76, 12.95 plus or minus 10.05, and 12.09 plus or minus 9.09.

Cohort 4: Primary school students after the Wenchuan earthquake. Wave 1 age 10.97 plus or minus 0.98, 48.9 percent female. P T S S scores at 1 and 1.5 years later are 13.22 plus or minus 7.07 and 10.60 plus or minus 6.93.

Cohort 5: Middle school students after the Yaan earthquake. Wave 1 age 14.51 plus or minus 1.93, 56.1 percent female. P T S S scores at 0.5, 1, 1.5, and 2 years later are 14.21 plus or minus 8.34, 14.43 plus or minus 8.05, 13.68 plus or minus 8.93, and 12.81 plus or minus 7.50.

Cohort 6: Middle school students after the Yaan earthquake. Wave 1 age 13.93 plus or minus 1.65, 56.1 percent female. P T S S scores at 3.5, 4.5, and 5.5 years later are 12.87 plus or minus 9.62, 12.73 plus or minus 9.41, and 14.00 plus or minus 10.87.

Cohort 7: Primary school students after the Yaan earthquake. Wave 1 age 9.73 plus or minus 0.91, 49.8 percent female. P T S S scores at 0.5, 1, 1.5, and 2 years later are 11.75 plus or minus 7.15, 12.48 plus or minus 8.20, 11.39 plus or minus 8.06, and 8.63 plus or minus 7.53.

Cohort 8: Middle school students after the Jiuzhaigou earthquake. Wave 1 age 14.60 plus or minus 1.54, 59.0 percent female. P T S S scores at 10, 22, and 27 months later are 28.91 plus or minus 13.79, 25.36 plus or minus 14.28, and 26.08 plus or minus 14.05.

Navigate back to Table 2..

### Table 3. Long description

The table is divided into eight cohorts across three earthquake events. For each cohort, the network temperature is normalized to 1 at the first time point.

* Cohort 1 (Wenchuan, Middle School): Time points at 1, 1.5, 2, and 2.5 years. Temperatures are 1, 1.03, 0.96, and 0.79. Average annual decline V sub T is 0.14.

* Cohort 2 (Wenchuan, Middle School): Time points at 3.5, 4.5, and 5.5 years. Temperatures are 1, 0.89, and 0.58. V sub T is 0.21.

* Cohort 3 (Wenchuan, Middle School): Time points at 8.5, 9.5, 10, and 10.5 years. Temperatures are 1, 0.92, 0.84, and 0.64. V sub T is 0.18.

* Cohort 4 (Wenchuan, Primary School): Time points at 1 and 1.5 years. Temperatures are 1 and 0.88. V sub T is 0.24.

* Cohort 5 (Yaan, Middle School): Time points at 0.5, 1, 1.5, and 2 years. Temperatures are 1, 0.89, 0.82, and 0.61. V sub T is 0.26.

* Cohort 6 (Yaan, Middle School): Time points at 3.5, 4.5, and 5.5 years. Temperatures are 1, 0.94, and 0.69. V sub T is 0.15.

* Cohort 7 (Yaan, Primary School): Time points at 0.5, 1, 1.5, and 2 years. Temperatures are 1, 0.87, 0.79, and 0.55. V sub T is 0.30.

* Cohort 8 (Jiuzhaigou, Middle School): Time points at 10, 22, and 27 months. Temperatures are 1, 0.97, and 0.68. V sub T is 0.23.

Navigate back to Table 3..

### Figure 1. Long description

Section A, titled Network Temperature, contains eight line graphs labeled Cohort 1 through Cohort 8. Each graph plots Network Temperature on the y-axis against Time Points on the x-axis.

* Cohort 1 (Middle School, Wenchuan): Shows a slight rise then a steep decline from 1 year to 2.5 years later.

* Cohort 2 (Middle School, Wenchuan): Shows a steady linear decline from 3.5 to 4.5 years later.

* Cohort 3 (Middle School, Wenchuan): Shows a steady decline from 8.5 to 10.5 years later.

* Cohort 4 (Primary School, Wenchuan): Shows a linear decline from 1 year to 1.5 years later.

* Cohort 5 (Middle School, Yaan): Shows a steady decline from 0.5 to 2 years later.

* Cohort 6 (Middle School, Yaan): Shows a steady decline from 3.5 to 5.5 years later.

* Cohort 7 (Primary School, Yaan): Shows a steady decline from 0.5 to 2 years later.

* Cohort 8 (Middle School, Jiuzhaigou): Shows a stable period followed by a very sharp decline between 22 and 27 months later.

All graphs in Section A feature error bars representing 95 percent confidence intervals.

Section B, titled Network Structure, presents the PTSD symptom network diagrams for Cohorts 1 through 8. For Cohorts 3, 6, and 8, the networks comprise nodes labeled from PTSD01 to PTSD20, whereas the remaining cohorts include nodes from PTSD01 to PTSD17. The thickness of each edge is proportional to the strength of the association between the two connected nodes/symptoms. Positive edges are depicted as solid blue lines, and negative edges as dashed red lines.

Navigate back to Figure 1..
