# Supplementary material for: Changes in the network temperature of posttraumatic stress disorder symptoms among children and adolescents following earthquakes
Source: Psychol Med. 2026 Jul 30;56:e242. doi: 10.1017/S0033291726105303 (PMC13439248; doi:10.1017/S0033291726105303)
Supplement: Supplementary file 1 [file S0033291726105303sup001.docx]

**Table S1**

*Detailed Information of the Three Earthquakes.*

| **Earthquake Name** | **Time** | **Location** | **Magnitude** | **Depth** | **Fatalities** | **Missing** | **Injured** |
| --- | --- | --- | --- | --- | --- | --- | --- |
| Wenchuan Earthquake | 12 May 2008 at 14:28:04 local time | near Yingxiu Town, Wenchuan County, Sichuan Province, China | moment magnitude of 8.0 | 14 km | 69,227 | 17,923 | 374,643 |
| Yaan Earthquake | 20 April 2013 at 08:02 local time | Lushan County, Yaan Prefecture, Sichuan Province, China (near Xichuan Village, Shuangshi Town) | moment magnitude of 7.0 | 13 km | 196 | 21 | 11,470 |
| Jiuzhaigou Earthquake | 8 August 2017 at 21:19:46 local time | near Bimang Village, Jiuzhaigou County, Sichuan Province, China | moment magnitude of 7.0 | 20 km | 25 | 6 | 525 |

**Table S2**

*Model Fit Results for Networks (Including Imputed Cases).*

(A) Middle School Students after the Wenchuan Earthquake (Cohort 1)

| model | DF | AIC | BIC | RMSEA | Chisq | Chisq_diff | DF_diff | p_value |
| --- | --- | --- | --- | --- | --- | --- | --- | --- |
| **6. equal networks and thresholds (sparse)** | **525** | **35805.79** | **36309.22** | **0.028** | **775.83** | **107.05** | **51** | **< 0.0001** |
| 8. all parameters equal (sparse) | 527 | 36102.85 | 36594.71 | 0.042 | 1076.89 | 301.06 | 2 | < 0.0001 |
| 4. equal networks (sparse) | 474 | 35800.73 | 36599.28 | 0.026 | 668.78 | 252.44 | 15 | < 0.0001 |
| 5. equal networks and thresholds (dense) | 456 | 35785.02 | 36687.73 | 0.024 | 617.07 | 108.47 | 51 | < 0.0001 |
| 7. all parameters equal (dense) | 459 | 36083.18 | 36968.52 | 0.041 | 921.22 | 304.15 | 3 | < 0.0001 |
| 3. equal networks (dense) | 405 | 35778.56 | 36976.37 | 0.021 | 508.6 | 54.34 | 45 | 0.16 |
| 2. all parameters free (sparse) | 360 | 35814.21 | 37272.42 | 0.021 | 454.26 | 454.26 | 360 | 0.00054 |
| 1. all parameters free (dense) | 0 | 36079.96 | 39621.32 | ~0 | | | | |

(B) Middle School Students after the Wenchuan Earthquake (Cohort 2)

| model | DF | AIC | BIC | RMSEA | Chisq | Chisq_diff | DF_diff | p_value |
| --- | --- | --- | --- | --- | --- | --- | --- | --- |
| **6. equal networks and thresholds (sparse)** | **395** | **15723.56** | **16037.4** | **0.039** | **592.49** | **57.87** | **34** | **0.0065** |
| 4. equal networks (sparse) | 361 | 15733.69 | 16214.25 | 0.038 | 534.62 | 276.23 | 55 | < 0.0001 |
| 2. all parameters free (sparse) | 294 | 15638.38 | 16447.5 | 0.011 | 305.32 | 41.73 | 24 | 0.014 |
| 8. all parameters equal (sparse) | 395 | 16146.68 | 16460.52 | 0.069 | 1015.61 | 423.12 | 0 | < 0.0001 |
| 5. equal networks and thresholds (dense) | 304 | 15707.45 | 16467.53 | 0.03 | 394.38 | 89.06 | 10 | < 0.0001 |
| 3. equal networks (dense) | 270 | 15728.11 | 16654.92 | 0.029 | 347.04 | 347.04 | 270 | 0.0011 |
| 7. all parameters equal (dense) | 306 | 16119.92 | 16870.19 | 0.07 | 810.85 | 416.47 | 2 | < 0.0001 |
| 1. all parameters free (dense) | 0 | 15921.07 | 18171.89 | ~0 | | | | |

(C) Middle School Students after the Wenchuan Earthquake (Cohort 3)

| model | DF | AIC | BIC | RMSEA | Chisq | Chisq_diff | DF_diff | p_value |
| --- | --- | --- | --- | --- | --- | --- | --- | --- |
| **6. equal networks and thresholds (sparse)** | **720** | **87840.86** | **88620.7** | **0.028** | **1429.27** | **1212.5** | **9** | **< 0.0001** |
| 4. equal networks (sparse) | 662 | 87623.06 | 88779.82 | 0.023 | 1095.47 | 1727.61 | 32 | < 0.0001 |
| 5. equal networks and thresholds (dense) | 627 | 87869.22 | 89253.43 | 0.029 | 1271.63 | 342.12 | 60 | < 0.0001 |
| 3. equal networks (dense) | 567 | 87647.1 | 89421.22 | 0.023 | 929.5 | 334.44 | 78 | < 0.0001 |
| 2. all parameters free (sparse) | 489 | 87468.66 | 89749.68 | 0.013 | 595.07 | 595.07 | 489 | 0.0007 |
| 8. all parameters equal (sparse) | 711 | 89071.36 | 89909.69 | 0.047 | 2641.77 | 1546.3 | 49 | < 0.0001 |
| 7. all parameters equal (dense) | 630 | 89414.68 | 90779.39 | 0.053 | 2823.08 | 1551.46 | 3 | < 0.0001 |
| 1. all parameters free (dense) | 0 | 87851.6 | 93310.44 | ~0 | | | | |

(D) Primary School Students after the Wenchuan Earthquake (Cohort 4)

| model | DF | AIC | BIC | RMSEA | Chisq | Chisq_diff | DF_diff | p_value |
| --- | --- | --- | --- | --- | --- | --- | --- | --- |
| **4. equal networks (sparse)** | **211** | **30826.51** | **31331.65** | **0.029** | **341.06** | **95.55** | **36** | **<0.0001** |
| 6. equal networks and thresholds (sparse) | 227 | 30915.74 | 31335.8 | 0.037 | 462.29 | 121.23 | 16 | <0.0001 |
| 8. all parameters equal (sparse) | 229 | 30929.4 | 31338.82 | 0.038 | 479.95 | 17.65 | 2 | 0.00015 |
| 2. all parameters free (sparse) | 175 | 30802.96 | 31499.51 | 0.023 | 245.5 | 54.03 | 22 | 0.00016 |
| 3. equal networks (dense) | 135 | 30798.95 | 31708.19 | 0.016 | 161.5 | 161.5 | 135 | 0.06 |
| 7. all parameters equal (dense) | 153 | 30900.99 | 31714.52 | 0.036 | 299.54 | 4.73 | 1 | 0.03 |
| 5. equal networks and thresholds (dense) | 152 | 30898.26 | 31717.11 | 0.035 | 294.81 | 133.31 | 17 | <0.0001 |
| 1. all parameters free (dense) | 0 | 30907.45 | 32534.52 | ~0 | | | | |

(E) Middle School Students after the Yaan Earthquake (Cohort 5)

| model | DF | AIC | BIC | RMSEA | Chisq | Chisq_diff | DF_diff | p_value |
| --- | --- | --- | --- | --- | --- | --- | --- | --- |
| **6. equal networks and thresholds (sparse)** | **531** | **32577.75** | **33031.91** | **0.041** | **990.11** | **184.42** | **53** | **< 0.0001** |
| 4. equal networks (sparse) | 478 | 32499.33 | 33250.65 | 0.037 | 805.69 | 597 | 19 | < 0.0001 |
| 5. equal networks and thresholds (dense) | 456 | 32542.56 | 33417.24 | 0.039 | 804.92 | 178.53 | 51 | < 0.0001 |
| 3. equal networks (dense) | 405 | 32466.03 | 33626.66 | 0.033 | 626.4 | 158.16 | 51 | < 0.0001 |
| 8. all parameters equal (sparse) | 531 | 33196.87 | 33651.02 | 0.064 | 1609.23 | 619.11 | 0 | < 0.0001 |
| 2. all parameters free (sparse) | 354 | 32409.87 | 33856.45 | 0.025 | 468.23 | 468.23 | 354 | < 0.0001 |
| 7. all parameters equal (dense) | 459 | 33134.33 | 33992.18 | 0.064 | 1402.69 | 597.76 | 3 | < 0.0001 |
| 1. all parameters free (dense) | 0 | 32649.64 | 36081.05 | ~0 | | | | |

(F) Middle School Students after the Yaan Earthquake (Cohort 6)

| model | DF | AIC | BIC | RMSEA | Chisq | Chisq_diff | DF_diff | p_value |
| --- | --- | --- | --- | --- | --- | --- | --- | --- |
| **6. equal networks and thresholds (sparse)** | **518** | **49861.95** | **50536.77** | **0.027** | **906.15** | **840.65** | **3** | **<0.0001** |
| 4. equal networks (sparse) | 477 | 49808.72 | 50730.57 | 0.025 | 770.92 | 977.69 | 57 | <0.0001 |
| 5. equal networks and thresholds (dense) | 418 | 49892.13 | 51169.47 | 0.027 | 736.33 | 125.78 | 40 | <0.0001 |
| 3. equal networks (dense) | 378 | 49846.35 | 51364.7 | 0.025 | 610.55 | 144.19 | 20 | <0.0001 |
| 2. all parameters free (sparse) | 358 | 49742.16 | 51381.02 | 0.017 | 466.37 | 466.37 | 358 | <0.0001 |
| 8. all parameters equal (sparse) | 515 | 50708.6 | 51401.5 | 0.048 | 1746.8 | 975.88 | 38 | <0.0001 |
| 7. all parameters equal (dense) | 420 | 50900.41 | 52165.7 | 0.056 | 1748.61 | 1012.28 | 2 | <0.0001 |
| 1. all parameters free (dense) | 0 | 49991.8 | 53787.67 | ~0 | | | | |

(G) Primary School Students after the Yaan Earthquake (Cohort 7)

| model | DF | AIC | BIC | RMSEA | Chisq | Chisq_diff | DF_diff | p_value |
| --- | --- | --- | --- | --- | --- | --- | --- | --- |
| 6. equal networks and thresholds (sparse) | 536 | 21971.02 | 22358.63 | 0.055 | 1036.14 | 244.58 | 52 | < 0.0001 |
| **4. equal networks (sparse)** | **484** | **21830.44** | **22483.24** | **0.046** | **791.55** | **637.26** | **25** | **< 0.0001** |
| 5. equal networks and thresholds (dense) | 456 | 21993.14 | 22788.74 | 0.057 | 898.25 | 252.88 | 51 | < 0.0001 |
| 2. all parameters free (sparse) | 387 | 21677.46 | 22824.96 | 0.022 | 444.57 | 444.57 | 387 | 0.023 |
| 8. all parameters equal (sparse) | 536 | 22500.52 | 22888.13 | 0.08 | 1565.63 | 529.5 | 0 | < 0.0001 |
| 3. equal networks (dense) | 405 | 21842.26 | 22897.97 | 0.044 | 645.37 | 200.8 | 18 | < 0.0001 |
| 7. all parameters equal (dense) | 459 | 22517.7 | 23298.01 | 0.084 | 1428.81 | 530.57 | 3 | < 0.0001 |
| 1. all parameters free (dense) | 0 | 22006.89 | 25128.11 | ~0 | | | | |

(H) Middle School Students after the Jiuzhaigou Earthquake (Cohort 8)

| model | DF | AIC | BIC | RMSEA | Chisq | Chisq_diff | DF_diff | p_value |
| --- | --- | --- | --- | --- | --- | --- | --- | --- |
| **6. equal networks and thresholds (sparse)** | **520** | **43423.45** | **44090.03** | **0.034** | **1165.43** | **933.04** | **8** | **<0.0001** |
| 4. equal networks (sparse) | 479 | 43341.54 | 44256.59 | 0.032 | 1001.53 | 1334.3 | 59 | <0.0001 |
| 5. equal networks and thresholds (dense) | 418 | 43380.02 | 44664.72 | 0.034 | 918.01 | 147.07 | 40 | <0.0001 |
| 2. all parameters free (sparse) | 364 | 43061.93 | 44673.87 | 0.018 | 491.92 | 491.92 | 364 | <0.0001 |
| 3. equal networks (dense) | 378 | 43312.94 | 44840.04 | 0.031 | 770.93 | 279.01 | 14 | <0.0001 |
| 8. all parameters equal (sparse) | 512 | 44372.48 | 45087.55 | 0.054 | 2098.47 | 1096.94 | 33 | <0.0001 |
| 7. all parameters equal (dense) | 420 | 44793.84 | 46066.43 | 0.066 | 2335.83 | 1417.83 | 2 | <0.0001 |
| 1. all parameters free (dense) | 0 | 43298.01 | 47115.75 | ~0 | | | | |

**Table S3**

*Model Fit Results for Networks (Non-imputed Cases).*

(A) Middle School Students after the Wenchuan Earthquake (Cohort 1)

| model | DF | AIC | BIC | RMSEA | Chisq | Chisq_diff | DF_diff | p_value |
| --- | --- | --- | --- | --- | --- | --- | --- | --- |
| 8. all parameters equal (sparse) | 557 | 10148.41 | 10388.37 | 0.06 | 849.96 | 14.01 | 3 | 0.0029 |
| 6. equal networks and thresholds (sparse) | 554 | 10140.4 | 10393.45 | 0.059 | 835.94 | 141.62 | 53 | <0.0001 |
| 4. equal networks (sparse) | 501 | 10104.78 | 10589.07 | 0.052 | 694.32 | 126.72 | 42 | <0.0001 |
| 7. all parameters equal (dense) | 459 | 10062.06 | 10729.6 | 0.04 | 567.61 | 8.13 | 3 | 0.043 |
| **5. equal networks and thresholds (dense)** | **456** | **10059.93** | **10740.56** | **0.04** | **559.47** | **78.14** | **51** | **0.0086** |
| 2. all parameters free (sparse) | 403 | 9994.76 | 10906.63 | ~0 | 388.3 | 388.3 | 403 | 0.69 |
| 3. equal networks (dense) | 405 | 10083.79 | 10986.94 | 0.036 | 481.34 | 93.03 | 2 | <0.0001 |
| 1. all parameters free (dense) | 0 | 10412.45 | 13082.63 | ~0 | | | | |

(B) Middle School Students after the Wenchuan Earthquake (Cohort 2)

| model | DF | AIC | BIC | RMSEA | Chisq | Chisq_diff | DF_diff | p_value |
| --- | --- | --- | --- | --- | --- | --- | --- | --- |
| 6. equal networks and thresholds (sparse) | 404 | 7686.85 | 7907.87 | 0.034 | 466.14 | 38.84 | 34 | 0.26 |
| 8. all parameters equal (sparse) | 406 | 7695.89 | 7908.87 | 0.036 | 479.18 | 13.04 | 2 | 0.0015 |
| **4. equal networks (sparse)** | **370** | **7716.01** | **8073.67** | **0.034** | **427.31** | **119.3** | **50** | **< 0.0001** |
| 2. all parameters free (sparse) | 320 | 7696.71 | 8255.3 | ~0 | 308.01 | 19.79 | 14 | 0.14 |
| 7. all parameters equal (dense) | 306 | 7744.5 | 8359.35 | 0.023 | 327.79 | 10.67 | 2 | 0.0048 |
| 5. equal networks and thresholds (dense) | 304 | 7737.83 | 8360.72 | 0.018 | 317.13 | 39.37 | 34 | 0.24 |
| 3. equal networks (dense) | 270 | 7766.47 | 8525.98 | 0.014 | 277.76 | 277.76 | 270 | 0.36 |
| 1. all parameters free (dense) | 0 | 8028.71 | 9873.24 | ~0 | | | | |

(C) Middle School Students after the Wenchuan Earthquake (Cohort 3)

| model | DF | AIC | BIC | RMSEA | Chisq | Chisq_diff | DF_diff | p_value |
| --- | --- | --- | --- | --- | --- | --- | --- | --- |
| **6. equal networks and thresholds (sparse)** | **745** | **38642.09** | **39168.9** | **0.027** | **1010.15** | **103.7** | **58** | **0.00021** |
| 8. all parameters equal (sparse) | 748 | 38754.96 | 39265.14 | 0.033 | 1129.03 | 118.88 | 3 | <0.0001 |
| 4. equal networks (sparse) | 687 | 38654.39 | 39502.83 | 0.026 | 906.46 | 53.9 | 57 | 0.59 |
| 5. equal networks and thresholds (dense) | 627 | 38604.48 | 39785.65 | 0.019 | 736.55 | 113.1 | 60 | <0.0001 |
| 7. all parameters equal (dense) | 630 | 38714.49 | 39879.02 | 0.027 | 852.56 | 116.01 | 3 | <0.0001 |
| 3. equal networks (dense) | 567 | 38611.39 | 40125.28 | 0.015 | 623.46 | 24.85 | 21 | 0.25 |
| 2. all parameters free (sparse) | 546 | 38628.54 | 40258.88 | 0.014 | 598.61 | 598.61 | 546 | 0.059 |
| 1. all parameters free (dense) | 0 | 39121.93 | 43780.06 | ~0 | | | | |

(D) Primary School Students after the Wenchuan Earthquake (Cohort 4)

| model | DF | AIC | BIC | RMSEA | Chisq | Chisq_diff | DF_diff | p_value |
| --- | --- | --- | --- | --- | --- | --- | --- | --- |
| **4. equal networks (sparse)** | **211** | **30826.51** | **31331.65** | **0.029** | **341.06** | **95.55** | **36** | **<0.0001** |
| 6. equal networks and thresholds (sparse) | 227 | 30915.74 | 31335.8 | 0.037 | 462.29 | 121.23 | 16 | <0.0001 |
| 8. all parameters equal (sparse) | 229 | 30929.4 | 31338.82 | 0.038 | 479.95 | 17.65 | 2 | 0.00015 |
| 2. all parameters free (sparse) | 175 | 30802.96 | 31499.51 | 0.023 | 245.5 | 54.03 | 22 | 0.00016 |
| 3. equal networks (dense) | 135 | 30798.95 | 31708.19 | 0.016 | 161.5 | 161.5 | 135 | 0.06 |
| 7. all parameters equal (dense) | 153 | 30900.99 | 31714.52 | 0.036 | 299.54 | 4.73 | 1 | 0.03 |
| 5. equal networks and thresholds (dense) | 152 | 30898.26 | 31717.11 | 0.035 | 294.81 | 133.31 | 17 | <0.0001 |
| 1. all parameters free (dense) | 0 | 30907.45 | 32534.52 | ~0 | | | | |

(E) Middle School Students after the Yaan Earthquake (Cohort 5)

| model | DF | AIC | BIC | RMSEA | Chisq | Chisq_diff | DF_diff | p_value |
| --- | --- | --- | --- | --- | --- | --- | --- | --- |
| **6. equal networks and thresholds (sparse)** | **548** | **12501.93** | **12790.59** | **0.047** | **751.7** | **95.96** | **52** | **0.0002** |
| 8. all parameters equal (sparse) | 552 | 12533.25 | 12803.86 | 0.051 | 791.02 | 39.31 | 4 | < 0.0001 |
| 4. equal networks (sparse) | 496 | 12509.97 | 13033.16 | 0.044 | 655.74 | 75.58 | 37 | 0.00019 |
| 5. equal networks and thresholds (dense) | 456 | 12493.72 | 13197.32 | 0.037 | 559.49 | 102.96 | 36 | < 0.0001 |
| 7. all parameters equal (dense) | 459 | 12508.39 | 13198.46 | 0.04 | 580.16 | 20.67 | 3 | 0.00012 |
| 2. all parameters free (sparse) | 420 | 12462.77 | 13328.74 | 0.023 | 456.54 | 14.37 | 15 | 0.50 |
| 3. equal networks (dense) | 405 | 12507.13 | 13440.76 | 0.031 | 470.9 | 470.9 | 405 | 0.013 |
| 1. all parameters free (dense) | 0 | 12846.23 | 15606.51 | ~0 | | | | |

(F) Middle School Students after the Yaan Earthquake (Cohort 6)

| model | DF | AIC | BIC | RMSEA | Chisq | Chisq_diff | DF_diff | p_value |
| --- | --- | --- | --- | --- | --- | --- | --- | --- |
| 8. all parameters equal (sparse) | 546 | 18226.8 | 18630.2 | 0.04 | 807.93 | 1.23 | 1 | 0.27 |
| **6. equal networks and thresholds (sparse)** | **545** | **18227.56** | **18635.77** | **0.04** | **806.69** | **62.08** | **40** | **0.014** |
| 4. equal networks (sparse) | 505 | 18245.49 | 18845.79 | 0.04 | 744.61 | 204.88 | 85 | <0.0001 |
| 2. all parameters free (sparse) | 418 | 18139.96 | 19158.07 | 0.019 | 465.09 | 17.98 | 40 | 1 |
| 5. equal networks and thresholds (dense) | 418 | 18171.76 | 19189.86 | 0.025 | 496.88 | 31.79 | 0 | <0.0001 |
| 7. all parameters equal (dense) | 420 | 18210.61 | 19219.11 | 0.031 | 539.74 | 42.85 | 2 | <0.0001 |
| 3. equal networks (dense) | 378 | 18201.99 | 19412.19 | 0.025 | 447.11 | 447.11 | 378 | 0.0082 |
| 1. all parameters free (dense) | 0 | 18510.87 | 21536.38 | ~0 | | | | |

(G) Primary School Students after the Yaan Earthquake (Cohort 7)

| model | DF | AIC | BIC | RMSEA | Chisq | Chisq_diff | DF_diff | p_value |
| --- | --- | --- | --- | --- | --- | --- | --- | --- |
| 8. all parameters equal (sparse) | 555 | 10762.44 | 11005.78 | 0.061 | 823.26 | 24.05 | 4 | < 0.0001 |
| 6. equal networks and thresholds (sparse) | 551 | 10746.39 | 11006.8 | 0.058 | 799.22 | 133.35 | 53 | < 0.0001 |
| 4. equal networks (sparse) | 498 | 10719.04 | 11205.72 | 0.051 | 665.86 | 38.37 | 39 | 0.5 |
| 2. all parameters free (sparse) | 435 | 10627.84 | 11383.47 | 0.015 | 448.67 | 31.14 | 30 | 0.41 |
| 7. all parameters equal (dense) | 459 | 10758.67 | 11411.85 | 0.053 | 627.5 | 10.47 | 3 | 0.015 |
| 5. equal networks and thresholds (dense) | 456 | 10754.2 | 11420.18 | 0.052 | 617.02 | 168.36 | 21 | < 0.0001 |
| **3. equal networks (dense)** | **405** | **10718.98** | **11602.69** | **0.037** | **479.81** | **479.81** | **405** | **0.0061** |
| 1. all parameters free (dense) | 0 | 11049.17 | 13661.86 | ~0 | | | | |

(H) Middle School Students after the Jiuzhaigou Earthquake (Cohort 8)

| model | DF | AIC | BIC | RMSEA | Chisq | Chisq_diff | DF_diff | p_value |
| --- | --- | --- | --- | --- | --- | --- | --- | --- |
| **6. equal networks and thresholds (sparse)** | **559** | **10222.34** | **10530.88** | **0.037** | **707.68** | **89.93** | **42** | **< 0.0001** |
| 8. all parameters equal (sparse) | 562 | 10272.36 | 10567.87 | 0.043 | 763.71 | 56.02 | 3 | < 0.0001 |
| 4. equal networks (sparse) | 517 | 10216.41 | 10707.47 | 0.032 | 617.75 | 197.6 | 76 | < 0.0001 |
| 2. all parameters free (sparse) | 441 | 10170.8 | 10992.13 | ~ 0 | 420.15 | 68.16 | 21 | < 0.0001 |
| 5. equal networks and thresholds (dense) | 418 | 10261.88 | 11183.15 | 0.024 | 465.22 | 79.35 | 40 | 0.00021 |
| 7. all parameters equal (dense) | 420 | 10280.96 | 11193.54 | 0.029 | 488.3 | 23.08 | 2 | < 0.0001 |
| 3. equal networks (dense) | 378 | 10262.53 | 11357.63 | 0.01 | 385.87 | 385.87 | 378 | 0.38 |
| 1. all parameters free (dense) | 0 | 10632.66 | 13370.41 | ~ 0 | | | | |

**Table S4**

*PTSD Network Temperature after Earthquakes (Non-imputed Cases).*

| Middle School Students after the Wenchuan Earthquake (Cohort 1) | Time points after the earthquake | 1 year later | 1.5 years later | 2 years later | 2.5 years later |
| --- | --- | --- | --- | --- | --- |
|  | Network temperature [95% CI] | 1 | 0.95 [0.89, 1.03] | 0.95 [0.88, 1.02] | 0.89 [0.84, 0.96] |
|  | Average annual decline in network temperature (V_T_) | 0.07 |  |  |  |
| Middle School Students after the Wenchuan Earthquake (Cohort 2) | Time points after the earthquake | 3.5 years later | 4.5 years later | 5.5 years later |  |
|  | Network temperature [95% CI] | 1 | 0.88 [0.81, 0.98] | 0.83 [0.76, 0.92] |  |
|  | Average annual decline in network temperature (V_T_) | 0.08 |  |  |  |
| Middle School Students after the Wenchuan Earthquake (Cohort 3) | Time points after the earthquake | 8.5 years later | 9.5 years later | 10 years later | 10.5 years later |
|  | Network temperature [95% CI] | 1 | 0.91 [0.87, 0.96] | 0.82 [0.79, 0.86] | 0.79 [0.76, 0.83] |
|  | Average annual decline in network temperature (V_T_) | 0.10 |  |  |  |
| Primary School Students after the Wenchuan Earthquake (Cohort 4) | Time points after the earthquake | 1 year later | 1.5 years later |  |  |
|  | Network temperature [95% CI] | 1 | 0.88 [0.84, 0.93] |  |  |
|  | Average annual decline in network temperature (V_T_) | 0.24 |  |  |  |
| Middle School Students after the Yaan Earthquake (Cohort 5) | Time points after the earthquake | 0.5 year later | 1 year later | 1.5 years later | 2 years later |
|  | Network temperature [95% CI] | 1 | 0.87 [0.81, 0.95] | 0.79 [0.74, 0.85] | 0.84 [0.78, 0.90] |
|  | Average annual decline in network temperature (V_T_) | 0.11 |  |  |  |
| Middle School Students after the Yaan Earthquake (Cohort 6) | Time points after the earthquake | 3.5 years later | 4.5 years later | 5.5 years later |  |
|  | Network temperature [95% CI] | 1 | 0.86 [0.81, 0.91] | 0.83 [0.78, 0.88] |  |
|  | Average annual decline in network temperature (V_T_) | 0.09 |  |  |  |
| Primary School Students after the Yaan Earthquake (Cohort 7) | Time points after the earthquake | 0.5 year later | 1 year later | 1.5 years later | 2 years later |
|  | Network temperature [95% CI] | 1 | 0.96 [0.87, 1.08] | 0.88 [0.80, 0.97] | 0.87 [0.79, 0.97] |
|  | Average annual decline in network temperature (V_T_) | 0.09 |  |  |  |
| Middle School Students after the Jiuzhaigou Earthquake (Cohort 8) | Time points after the earthquake | 10 months later | 22 months later | 27 months later |  |
|  | Network temperature [95% CI] | 1 | 0.96 [0.91, 1.02] | 0.88 [0.84, 0.93] |  |
|  | Average annual decline in network temperature (V_T_) | 0.08 |  |  |  |

**Table S5**

*PTSD Network Entropy after Earthquakes.*

| Middle School Students after the Wenchuan Earthquake (Cohort 1) | Time points after the earthquake | 1 year later | 1.5 years later | 2 years later | 2.5 years later |
| --- | --- | --- | --- | --- | --- |
|  | Entropy of the imputed case dataset | 8.17 | 8.49 | 7.70 | 5.23 |
|  | Entropy of the non-imputed case dataset | 8.87 | 8.48 | 8.42 | 7.85 |
| Middle School Students after the Wenchuan Earthquake (Cohort 2) | Time points after the earthquake | 3.5 years later | 4.5 years later | 5.5 years later |  |
|  | Entropy of the imputed case dataset | 9.67 | 9.05 | 4.77 |  |
|  | Entropy of the non-imputed case dataset | 9.69 | 9.08 | 8.74 |  |
| Middle School Students after the Wenchuan Earthquake (Cohort 3) | Time points after the earthquake | 8.5 years later | 9.5 years later | 10 years later | 10.5 years later |
|  | Entropy of the imputed case dataset | 10.84 | 10.10 | 9.24 | 5.52 |
|  | Entropy of the non-imputed case dataset | 11.15 | 10.52 | 9.68 | 9.30 |
| Primary School Students after the Wenchuan Earthquake (Cohort 4) | Time points after the earthquake | 1 year later | 1.5 years later |  |  |
|  | Entropy of the imputed case dataset | 10.27 | 10.07 |  |  |
|  | Entropy of the non-imputed case dataset | 10.27 | 10.07 |  |  |
| Middle School Students after the Yaan Earthquake (Cohort 5) | Time points after the earthquake | 0.5 year later | 1 year later | 1.5 years later | 2 years later |
|  | Entropy of the imputed case dataset | 9.81 | 9.09 | 8.43 | 4.89 |
|  | Entropy of the non-imputed case dataset | 9.93 | 9.26 | 8.63 | 9.01 |
| Middle School Students after the Yaan Earthquake (Cohort 6) | Time points after the earthquake | 3.5 years later | 4.5 years later | 5.5 years later |  |
|  | Entropy of the imputed case dataset | 10.04 | 9.33 | 4.99 |  |
|  | Entropy of the non-imputed case dataset | 10.94 | 9.74 | 9.41 |  |
| Primary School Students after the Yaan Earthquake (Cohort 7) | Time points after the earthquake | 0.5 year later | 1 year later | 1.5 years later | 2 years later |
|  | Entropy of the imputed case dataset | 10.35 | 10.13 | 9.67 | 5.44 |
|  | Entropy of the non-imputed case dataset | 9.98 | 10.08 | 9.71 | 9.26 |
| Middle School Students after the Jiuzhaigou Earthquake (Cohort 8) | Time points after the earthquake | 10 months later | 22 months later | 27 months later |  |
|  | Entropy of the imputed case dataset | 9.05 | 8.65 | 2.78 |  |
|  | Entropy of the non-imputed case dataset | 9.66 | 9.11 | 7.76 |  |

**Table S6**

*Node Names of PTSD Symptoms.*

| Labels | Node names |
| --- | --- |
| PTSS01 | Loss of interest in activities |
| PTSS02 | Negative beliefs about the future (hopes won’t come true) |
| PTSS03 | Avoidance of reminders (activities, people, places) |
| PTSS04 | Difficulty concentrating |
| PTSS05 | Sleep disturbance (difficulty falling or staying asleep) |
| PTSS06 | Nightmares |
| PTSS07 | Exaggerated startle response |
| PTSS08 | Intrusive, distressing memories or images |
| PTSS09 | Emotional distress when reminded of the event |
| PTSS10 | Emotional numbing |
| PTSS11 | Flashbacks (reliving the event) |
| PTSS12 | Physiological reactions when reminded |
| PTSS13 | Hypervigilance |
| PTSS14 | Inability to remember important aspects of the event |
| PTSS15 | Irritability or angry outbursts |
| PTSS16 | Feeling detached from others |
| PTSS17 | Avoidance of thoughts or feelings about the event |
| PTSS18 | Reckless behavior |
| PTSS19 | Blaming oneself or others without reason |
| PTSS20 | Inability to experience positive emotions |

**Figure S1**

*PTSD Symptoms Occurrence after Earthquakes (Including Imputed Cases).*

**Figure S2**

*The Proportion of the Mean PTSD Symptoms Score Relative to the Total Score after Earthquakes (Including Imputed Cases).*

**Figure S3**

*PTSD Network Temperature after Earthquakes (Non-imputed Cases).*

(A) Network Temperature

(B) Network Structure

*Note:* The error bars indicate 95% confidence intervals. See Table S6 for node names. Solid lines denote positive edges; dashed lines denote negative edges. When estimating network temperature changes across all cohorts, the Ising model with edge weights constrained to be equal across waves within each cohort provided the best fit to the data (see Table S3). Consequently, the displayed network structure is representative of all waves, as the edges are identical across time points.
